# Identification of biomarkers and prediction of upstream miRNAs in diabetic nephropathy

**DOI:** 10.3389/fendo.2023.1144331

**Published:** 2023-02-21

**Authors:** Dapeng Yin, Zhixin Guo, Xinyu Zhang

**Affiliations:** Department of Endocrinology, The Second Medical Hospital of Shanxi Medical University, Taiyuan, Shanxi, China

**Keywords:** diabetic nephropathy, MMP2, miRNA, bioinformatics, fibrosis

## Abstract

**Objective:**

To explore biomarkers of diabetic nephropathy (DN) and predict upstream miRNAs.

**Methods:**

The data sets GSE142025 and GSE96804 were obtained from Gene Expression Omnibus database. Subsequently, common differentially expressed genes (DEGs) of renal tissue in DN and control group were identified and protein-protein interaction network (PPI) was constructed. Hub genes were screened from in DEGs and made an investigation on functional enrichment and pathway research. Finally, the target gene was selected for further study. The receiver operating characteristic (ROC) curve was used to evaluate the diagnostic efficiency of target gene and predicted its upstream miRNAs.

**Results:**

130 common DEGs were obtained through analysis, and 10 Hub genes were further identified. The function of Hub genes was mainly related to extracellular matrix (ECM), collagen fibrous tissue, transforming growth factor (TGF) -β, advanced glycosylation end product (AGE) -receptor (RAGE) and so on. Research showed that the expression level of Hub genes in DN group was significantly higher than that in control group. (all P<0.05). The target gene matrix metalloproteinase 2 (MMP2) was selected for further study, and it was found to be related to the fibrosis process and the genes regulating fibrosis. Meanwhile, ROC curve analysis showed that MMP2 had a good predictive value for DN. miRNA prediction suggested that miR-106b-5p and miR-93-5p could regulate the expression of MMP2.

**Conclusion:**

MMP2 can be used as a biomarker for DN to participate in the pathogenesis of fibrosis, and miR-106b-5p and miR-93-5p may regulate the expression of MMP2 as upstream signals.

## 1 Introduction

Diabetic nephropathy (DN) is a common microvascular complication of diabetes and a major cause of end-stage renal disease (ESRD) throughout the world (1). DN is characterized by glomerular basement membrane thickening, mesangial expansion and accumulation of matrix, and eventually progression to fibrosis of glomeruli and renal tubules. Its pathogenesis is very complex, involving advanced glycosylation end products (AGE), inflammation, oxidative stress, apoptosis, autophagy and various signaling mechanisms leading to extracellular matrix (ECM) deposition and renal interstitial fibrosis (2). Finally, it leads to the appearance of albuminuria and the progressive decrease of estimated glomerular filtration rate (eGFR). Therefore, new identification and therapeutic tools to improve DN are urgently needed.

miRNA is an endogenous non-coding RNA (about 22 nucleotides long) that usually targets the 3 ‘untranslated region (3’ -UTRs) of mRNA, resulting in post-transcriptional gene silencing (3). A large amount of evidence has shown that miRNA is involved in the occurrence of diabetes and its complications, such as DN, neuropathy, retinopathy, cardiomyopathy and wound healing. Zhong et al. found that miR-21 is highly expressed in the renal cortex of diabetes mice with microalbuminuria and positively regulated the expression of ECM by regulating transforming growth factor (TGF)- β, Nuclear factor kB (NF kB) and Smad homolog 7 (SMAD7) play a pathological role in renal fibrosis and inflammation (4). Therefore, regulation of miRNA is promising in the treatment of DN.

**Figure 1:**
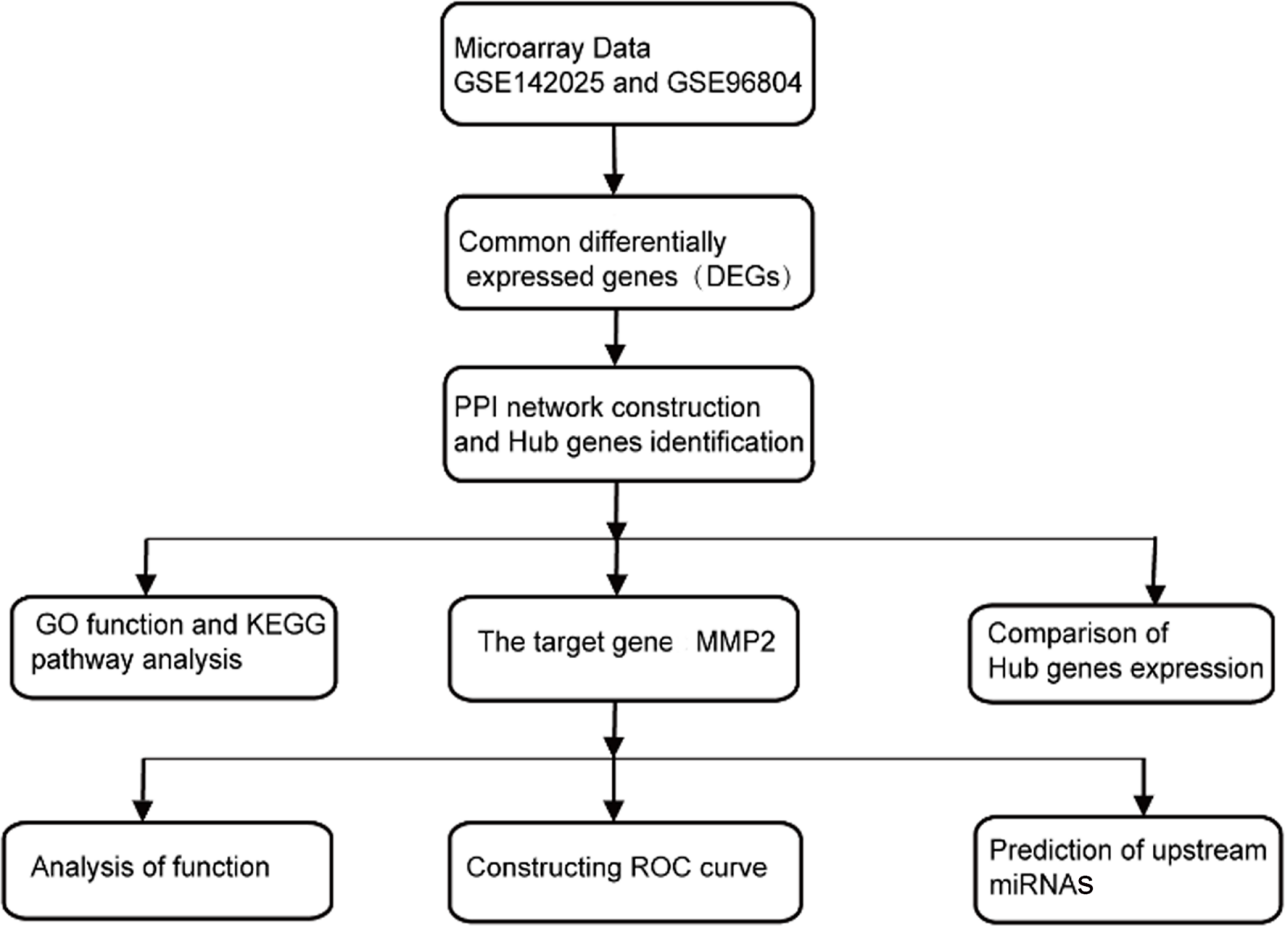
Work flow diagram includes data preparation, processing and analysis.

Bioinformatics, which research objects mainly focus on gene and protein, has played a vital role in the research of life science with its rapidly development in recent years. Although many mechanisms are involved in the pathogenesis of DN, the potential bioinformatics driving its pathogenesis is rarely fully elucidated. In this study, the gene expression data of DN kidney tissue from the Gene Expression Omnibus (GEO) was analyzed to predict the key genes causing renal fibrosis and explore their upstream miRNAs. This provides a new idea for the early diagnosis and treatment of DN. The specific working flow chart is shown in **Figure 1**.

## 2 Methods

### 2.1 Microarray data

The DN gene expression data sets were retrieved in the NCBI-GEO database (https://www.ncbi.nlm.nih.gov/geo/) using the keywords “diabetic nephropathy” and “diabetic kidney disease”. Finally, two renal tissue gene expression data sets GSE142025 and GSE96804 with relatively large sample size were finally obtained. GSE142025 is based on GPL20301 platform, including 27 cases of DN and 9 cases of normal control group (5). GSE96804 is based on GPL17586 platform, including 41 cases of DN and 20 cases of renal tissue control not affected by tumor (6). After downloading the gene expression matrix, the probe identification number (ID) was converted into gene symbol using R software (version 4.2.2). For multiple probes corresponding to a gene, the average expression value was taken as the gene expression value.

### 2.2 Differential expression analysis

Limma software package can perform differential expression analysis on RNA sequencing data and high-throughput data, which contains powerful functions of reading, standardizing and exploring data (7). The R software and the limma package in Bioconductor were used to identify differentially expressed genes (DEGs) between DN and control group. The ratio of gene expression between the two groups (fold change, FC) was calculated, and the logarithm base 2 (log2FC) and adjusted P-value were selected. In line with the adjusted P - value < 0.05, | log2FC | > 1 genes are considered to be DEGs. Negative values represent down- regulated gene, while positive values represent up-regulated gene. Use ggplot 2 package (R software) to make volcanic maps. Then, through the online website (http://bioinformatics.psb.ugent.be/webtools/Venn/) draw Venn diagram to identify common DEGs for subsequent analysis.

### 2.3 PPI network construction and hub gene identification

STRING( https://cn.string-db.org/) (version 11.5) is an online tool for interacting gene retrieval, which provides new insights into the molecular mechanism of disease occurrence by integrating protein interactions (8). Protein-protein interaction (PPI) networks for common DEGs were predicted by STRING. The confidence score (>0.40) was used as the screening criteria, and the results were visualized by the CytoScape software (version 3.8.0). CytoScape is an open source bioinformatics software platform for visualizing molecular interaction networks. CytoHubba is a tool for defining network topology to find Hub genes. Subsequently, MCC algorithm of CytoHubba plugin was used to select the top 10 genes with the highest node connection closeness as the Hub genes, and boxplot was drawn to show the expression level.

### 2.4 Biological function and pathway analysis

DAVID database (https://david.ncifcrf.gov/) (version DAVID2021), an online gene function classification tool, through the powerful aggregation algorithm, complete the annotation, visualization and integration for gene and protein function (9). Gene Ontology (GO) analysis is a bioinformatics method that annotates genes and their protein products. It is widely used in analyzing the functional similarity between genes and identifying biological functions and pathways related to diseases by high-throughput biological data analysis (10). The Kyoto Encyclopedia of Genes and Genomes (KEGG) is a public and comprehensive database for understanding the advanced functions of cells and organisms from genomics and other high-throughput data sets, integrating genomes, biological pathways, diseases, drugs and chemicals (11, 12). In order to understand the function of Hub genes, we used the DAVID database for GO functional analysis and KEGG pathway enrichment. Typically, GO analyses are annotated with biological processes (BP), cellular components (CC), and molecular functions (MF). P<0.05 was considered statistically significant. If there are more than 10 results, this study will only show the top 10 results in P-value ranking.

### 2.5 Genomic variation analysis and correlation analysis

The first gene ranked by MCC algorithm among Hub genes was selected as the target gene analysis. Each sample in the dataset GSE142025 was scored for fibrosis enrichment using the GSVA package in Bioconductor and R software, and the results were visualized using heat maps. Subsequently, the correlation between target genes and common fibrosis genes was analyzed by Pearson correlation. The genes related to fibrosis function were collected from the GSEA (http://www.gsea-msigdb.org/gsea/index.jsp) (version 4.3.2).

### 2.6 ROC curve analysis

In order to effectively distinguish patients with DN from the control group, we analyzed the ROC curve of the target gene using the pROC package (R software).

### 2.7 miRNA prediction

Encyclopedia of RNA Interactors (ENCORI) database (https://starbase.sysu.edu.cn/) is an open source network tool for studying the interactions among ncRNAs and RNA-RNA (13). Based on the ENCORI database, we predicted the upstream miRNAs of the Hub genes, and the screening results were jointly calculated by miRanda and Targetscan plugins. The results were presented in the network diagram by the CytoScape software.

### 2.8 Statistical analysis

Graphpad Prism (version 9.3.0) and R software were used for statistical analysis. The Hub genes expression analysis uses t test, and the pROC package (R software) was used for ROC curve analysis to calculate the area under the curve (AUC).

## 3 Results

### 3.1 Identification of DEGs

**Figure 2:**
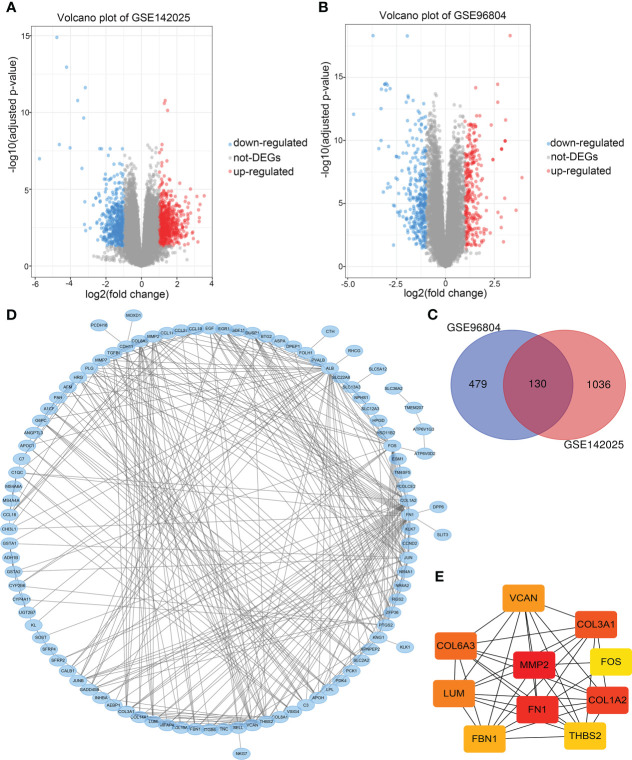
**(A)** Volcano maps of differentially expressed genes in data sets GSE1422025, **(B)** GSE96804. **(C)** Common differentially expressed genes in the two data sets. **(D)** PPI network of differentially expressed genes. **(E)** The PPI network of the top 10 Hub genes.

Take adjusted P-value<0.05, | log2FC |>1 as the screening criteria. 1166 DEGs were obtained from the GSE142025 dataset, including 660 up-regulated and 506 down-regulated genes. 609 DEGs were obtained from GSE96804 dataset, including 283 up-regulated and 326 down-regulated genes. The volcano maps were drawn separately to show the DEGs of two data sets (**Figures 2A, B**), and 130 common DEGs were identified by Venn diagram (**Figure 2C**). **Table 1** lists common DEGs, in which NPIPB5, FAM151A and NKG7 have opposite expressions in the two data sets are not listed.

**Table 1:** Differential expression genes identified from two data sets.

DEGs	Gene names
up-regulated	MMP2,MMP7,C1QC,SELL,CCL19,COL15A1,C3,PCDH18,FN1,MS4A6A, SFRP2,SVEP1,ITGB6,SLIT3,SFRP4,VSIG4,CDH11,FBN1,C7,COL8A1, ABCC9,COL3A1,CCL11,LUM,MOXD1,MFAP4,TNC,CCL18,INHBA,MS4A4A, IGFBP6,CPA3,COL14A1,AEBP1,THBS2,OLFML2B,CCL21,VCAN,ADH1B, CCND2,COL6A3,TGFBI,COL1A2,APOC1
down-regulated	XPNPEP2,BTG2,GSTA1,LINC00839,GDF15,HRG,NR4A2,CALB1,HSD11B2,UGT2B,HES1, A1CF,FOLH1B,GSTA2,ANGPTL3,KL,C9orf66, ATP6V1G3, CHI3L1,NPHS1,EGR1,SLC22A8,ALDH6A1,ZFP36,EGF,ASPA, CTH, PCK1, BRE-AS1,PTGS2,DUSP1, SOST, WDR49,AFM,HPGD,RHCG, CYP4A11, NPIPB3,TMEM207,TMEM178A,PDK4, DPP6,GADD45B,MRO,RGS2, GIPC2, SLC12A3,SLC5A12,KLK7,CPXM1,NELL1,NR4A1, APOH,FOS,JUNB, PAH, RNF152,PVALB,TMEM52B,RASD1,SLC36A2,KNG1,DPEP1,PLG, LPL, ALB, ATP6V0D2,HSPA1B,SLC2A2,CLDN8,CYP2B6,KLK1,G6PC, TREM1, ESM1, SLC13A3,ERRFI1,JUN,PCOLCE2,FAM180A,TM4SF5,FMN2,PRODH2

### 3.2 PPI network and hub gene screening

PPI network analysis was performed on 130 common DEGs using STRING and Cytoscape for visualization (**Figure 2D**). The CytoHubba plugin was used for PPI network correlation analysis, and 10 Hub genes were identified according to MCC algorithm (**Figure 2E**). In order of rank, they were MMP2, FN1, COL1A2, COL3A1, COL6A3, LUM, VCAN, FBN1, THBS2 and FOS. Finally, MMP2, which was ranked first, was selected as the target gene for further analysis.

### 3.3 GO function and KEGG pathway enrichment analysis

**Figure 3:**
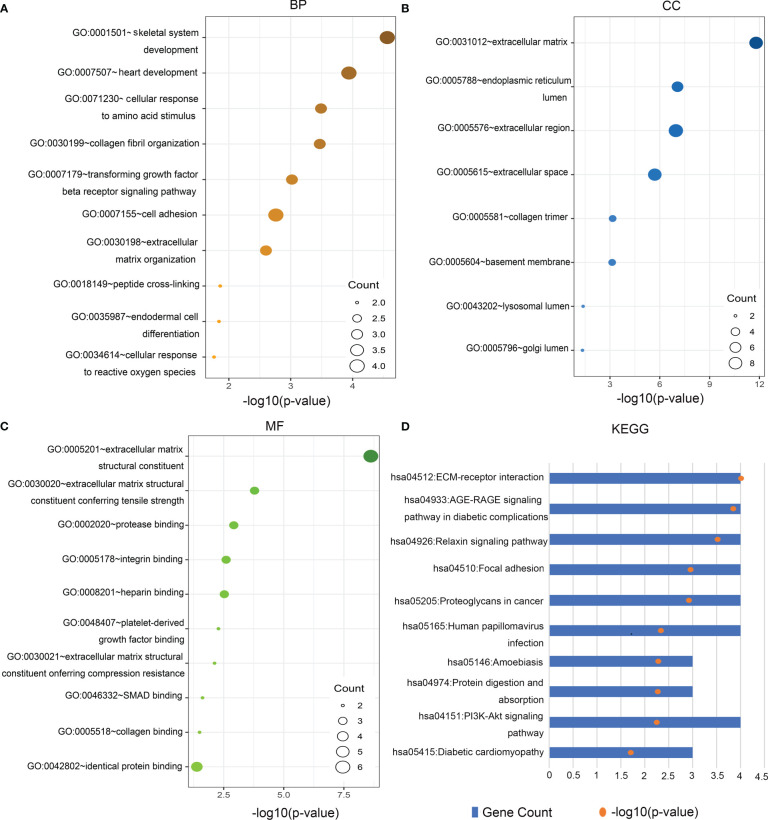
Gene Ontology (GO) function and Kyoto Encyclopedia of Genes and Genomes (KEGG) pathway analysis of Hub genes. **(A)** Biological Processes (BP), **(B)** cellular components (CC), **(C)** molecular functions (MF), **(D)** KEGG.

GO functional analysis of the Hub genes showed that biological processes were mainly enriched in skeletal system development, heart development, cellular response to amino acid stimulus, collagen fibril organization, TGF-β receptor signaling pathway, cell adhesion, extracellular matrix organization, cellular response to reactive oxygen species and other functions (**Figure 3A**). In terms of cell components, genes were mainly enriched in ECM, extracellular region, and organelles of endocellular structures (**Figure 3B**). At the molecular level, genes were enriched in ECM structural components, ECM structures related to tensile and compressive strength, protease binding, integrin binding and heparin binding (**Figure 3C**). KEGG pathway analysis showed that the Hub genes was enriched in ECM-receptor interaction, AGE-RAGE signaling pathway in diabetic complications, relaxin signaling pathway focal adhesion, PI3K-Akt signaling pathway, and so on (**Figure 3D**). (all P<0.05). These findings indicate that Hub gene is involved in the occurrence of DN and plays an important role in regulating ECM formation and fibrosis.

### 3.4 MMP2 participates in the development of fibrosis

**Figure 4:**
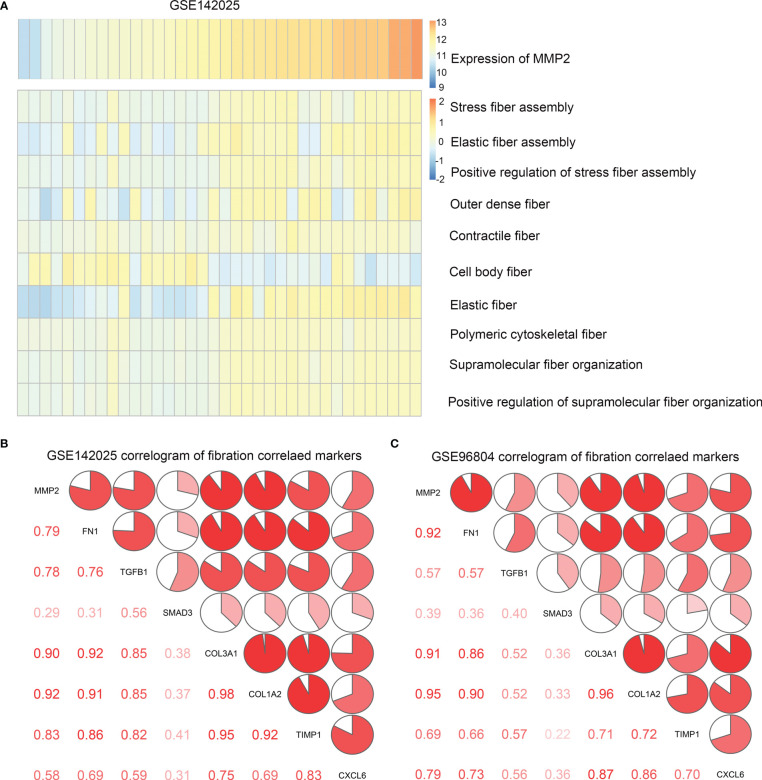
**(A)** Heat map of correlation between MMP2 expression and fibrosis function enrichment score. The samples were arranged in ascending order of MMP2 expression. **(B)** Correlation between MMP2 and fibrosis gene in data sets GSE142025, **(C)** GSE96804. Correlation coefficients are shown in the lower left corner. In the upper right, the correlation coefficients are also shown to be pie chart proportions.

Renal fibrosis is one of the main causes of DN, which is characterized by the activation and proliferation of fibroblasts and the deposition of ECM (14). Matrix metalloproteinase-2(MMP2) play an important role in renal fibrosis and DN (15). Therefore, we investigated the effect of MMP2 activation on fibrotic pathways and cytokine characteristics. GSVA was used to determine the enrichment fraction of the fibrosis process. The results showed that in the GSE142025 database, MMP2 expression was positively correlated with most fibrosis functions, including stress fiber assembly, elastic fiber assembly, outer dense fiber, contraction fiber and other functions (**Figure 4A**). The correlation was tested by Person correlation analysis. In both data sets, the correlation coefficients between MMP2 and common fibrosis genes were positive numbers (**Table 2**). It was shown that MMP2 was positively correlated with common fibrosis genes such as TGFB1, FN1 and CXCL6 (**Figures 4B, C**). These results support the hypothesis that MMP2 is involved in the regulation of fibrosis during the development of DN.

**Table 2:** Correlation analysis between MMP2 and common fibrosis genes.

Correlation Coefficient	FN1	TGFB1	SMAD3	COL3A1	COL1A2	TIMP1	CXCL6
MMP2 in GSE142025	0.79	0.78	0.29	0.90	0.92	0.83	0.58
MMP2 in GSE96804	0.92	0.57	0.39	0.91	0.95	0.69	0.79

### 3.5 The expression of Hub gene and its diagnostic value in DN

**Figure 5:**
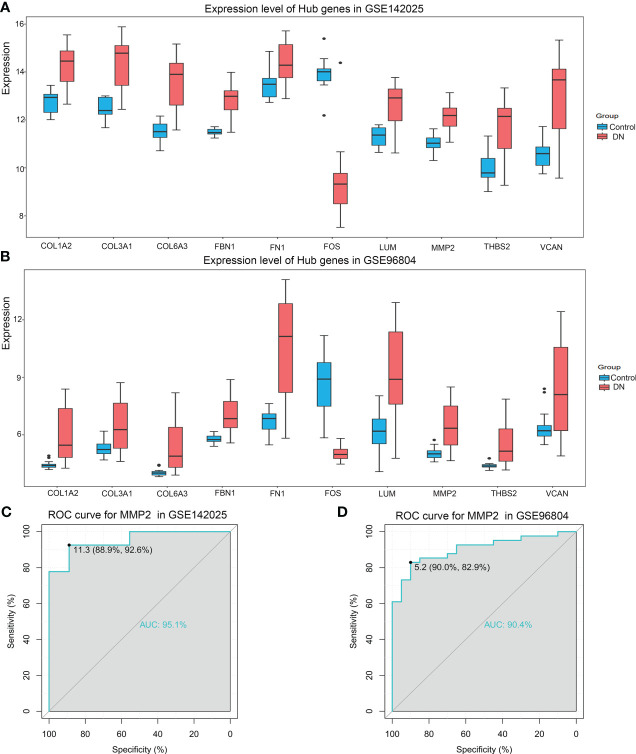
**(A)** Expression levels of hub genes in data sets GSE142025, **(B)** GSE96804, all P-value<0.05. **(C)** Receiver Operating Characteristic (ROC) curves of MMP2 in GSE142025, **(D)** GSE96804. The area under the curve (AUC) were 95.1% and 90.0%, respectively.

In the GSE142025 dataset, compared with the control group, the expressions of MMP2, FN1, COL1A2, COL3A1, COL6A3, LUM, VCAN, FBN1, THBS2 in DN renal tissue were up-regulated, while FOS expression was down-regulated (P<0.05). This result was verified in the GSE96804 dataset (**Figures 5A, B**). The target gene MMP2 was selected for ROC curve analysis, and the results show that AUC in the data sets GSE142025 and GSE96804 were 95.1% and 90.4% respectively (**Figures 5C, D**). This suggests that MMP2 is significantly enriched in DN and can be used as a potential biomarker to predict its occurrence.

### 3.6 Prediction of miRNA regulating MMP2

**Figure 6:**
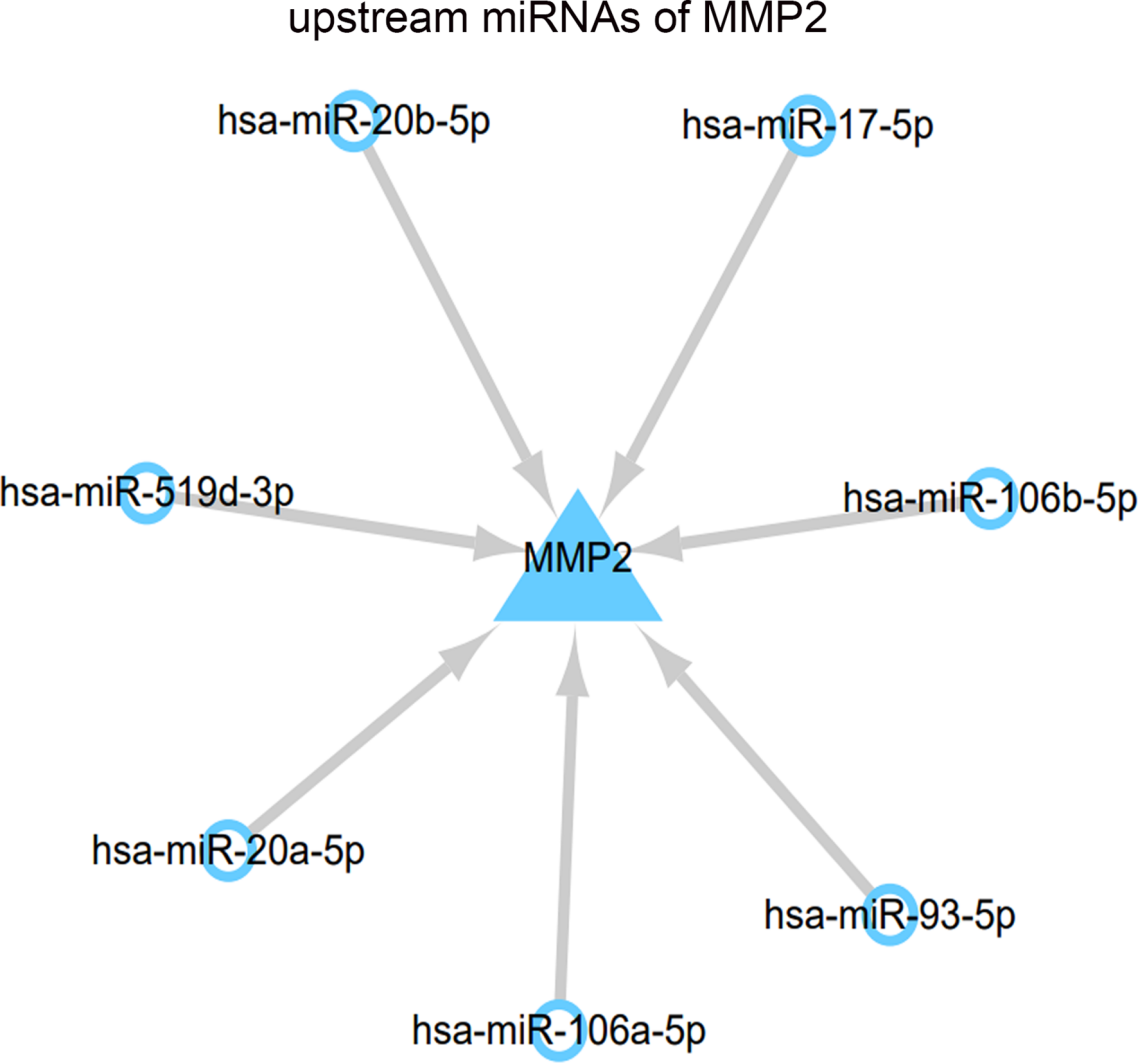
Regulatory network of MMP2 and upstream miRNAs.

In this study, the upstream miRNAs of MMP2 were predicted through ENCORI database, 13 miRNAs were predicted in TargetScan plugin, and 21 miRNAs were predicted in miRanda plugin. The intersection of the two algorithms was selected. Finally, the results showed that the upstream miRNAs of MMP2 included hsa-miR-17-5p, hsa-miR-20a-5p, hsa-miR-93-5p, hsa-miR-106a-5p, hsa-miR-106b-5p, and hsa-miR-106B-5P. hsa-miR-20b-5p and hsa-miR-519d-3p (**Figure 6**).

## 4 Discussion

DN is the most common microvascular complication of diabetes, increasing in incidence year on year and becomes the main cause of end-stage renal disease (16). At present, there are few studies on the genome of DN. Exploring its changes in the genome, analyzing the involved biological functions and predicting the possible molecular mechanisms will be helpful for the diagnosis and treatment of DN.

In this study, the common DEGs of DN and the control group were obtained based on the gene chip matrix, and 10 Hub genes were finally selected for GO functional analysis and KEGG pathway enrichment. It is found that the functions of Hub genes are mainly concentrated in “collagen fiber tissue, ECM components, TGF-β receptor signaling pathway” and other aspects. This is consistent with the previous study. Some scholars found that genes related to angiogenesis, ECM secretion and immune cell infiltration were involved in the occurrence of diseases through single cell sequencing of DN glomerulus. At the same time, this study revealed that although eGFR was relatively normal at the early stage of the disease, there were mild to moderate glomerulosclerosis and interstitial fibrosis (17). In DN patients and animal models, urinary TGF-β level was higher than control group, and plasma TGF-β1 level was closely correlated with the severity of renal dysfunction (18). Overproduction of TGF-β1 in the glomerular apparatus led to proteinuria, polyuria, decreased eGFR, and increased ECM deposition in the glomeruli, indicating that TGF- β participated in the occurrence of DN (19). These studies are basically consistent with the functions enriched by Hub genes, suggesting that these genes have diagnostic and therapeutic value for DN.

The enrichment of KEGG pathway showed significant enrichment in “ECM-receptor interaction, AGE-RAGE signaling pathway in diabetic complications, PI3K-Akt signaling pathway, adhesion plaque” and other aspects. It was found that AGE can induce collagen production, and inhibition of collagen production can significantly reduce the severity of DN. At the same time, AGE can activate TGF- β/Smad pathway and enhanced TGF- β Transcriptional activity. In addition, the crosstalk mechanism of AGE (RAGE)-ERK/p38MAKPs-Smad can activate Smad2/3 to play an important role in DN (19). This indicates that AGE-RAGE and TGF-β pathway related genes play an important role in the process of DN fibrosis. On the other hand, AGE-RAGE signaling pathway is related to the production of reactive oxygen species (ROS) and mitochondrial dysfunction. The inflammatory reaction mediated by NF-kB and PI3K-Akt signaling pathways triggered by ROS aggravates the renal injury in diabetes (20). Therefore, it is speculated that genes involved in the PI3K-Akt pathway and AGE-RAGE pathway have a hand in DN in the induction of oxidative stress and inflammation response.

For this reason, improving renal fibrosis plays a positive role in the treatment of DN. In this context, we aimed to further explore the related genes involved in fibrosis, finally selected the target gene MMP2 for further research, explored the miRNAs acting on this gene, and find a new therapeutic target to improve DN.

MMP2 plays a key role in the development of chronic kidney disease by remodeling the extracellular matrix, distorting the structure of the glomerular basement membrane, contributing to the development of tubulointerstitial fibrosis and leading to progressive kidney injury (15). The study found that the expression of MMP2 in renal tissue of DN patients increased (21), and the serum level of MMP-2 was significantly higher than that of normal healthy subjects (22). Serum creatinine, eGFR and proteinuria were remarkable correlated with serum MMP-2 levels in patients with DN (22). This was consistent with our research findings, the expression level of MMP2 in DN nephridial tissue was significantly higher than in the control group. At the same time, GSVA analysis indicated that MMP2 was bound up with the most fibrosis processes. Correlation analysis showed that the expression of MMP2 was positively correlated with common fibrosis genes such as TGFB1, FN1, CXCL6, etc. Based on the above results, we predicted that MMP2 could be used as a relevant target for the diagnosis of DN and drug intervention. Subsequently, ROC curve analysis verified that MMP2 could effectively distinguish patients with DN from the control group. The involvement of MMP2 in the occurrence of chronic kidney disease also contains other mechanisms, such as inducing the production of mitochondrial ROS, mitochondrial autophagy, activating systemic inflammatory response, and infiltration of renal monocytes, which cause structural abnormalities in the kidney (23). This requires us to further study its mechanism in DN.

miRNA is found to regulate the signaling pathways related to inflammation, autophagy, apoptosis and fibrosis, and participate in the occurrence of DN. It is reported that many miRNAs, like miR-21, miR-217, miR-216a, miR-200, miR-195, miR-451, miR-141, miR-93, miR-29, etc regulate DN signal transduction by combining with 3’UTR of target genes (24). Similarly, multiple drugs play a protective role in DN by regulating miRNA expression. Resveratrol upregulates autophagy and inhibits apoptosis by inhibiting the expression of miR-383-5p (25). It could also inhibit apoptosis by up regulating the expression of miR-18a-5p, thus having beneficial effects on the kidney (26). Hyperoside could cause epigenetic changes by down-regulation of mi-R21 and increase the level of MMP-9 protein (27). Through the above mechanism, hyperoside could regulate ECM and have an impact on the renal function of DN mice (28). For this reason, this study predicted the upstream miRNAs regulating MMP2, and identified that hsa-miR-17-5p, hsa-miR-20a-5p, hsa-miR-93-5p, hsa-miR-106a-5p, hsa-miR-106b-5p, hsa-miR-20b-5p and hsa-miR-519d-3p could regulate the expression of MMP2. Previous study has shown that miR-106b-5p is involved in the development of chronic thromboembolic pulmonary hypertension by negatively regulating the expression of MMP2 (29). miR-93-5p inhibits the proliferation, invasion and migration of tumor cells by targeting MMP2 in glioma (30). This provides a theoretical basis for miR-106b-5p and miR-93-5p to regulate MMP2 and thus delay the development of DN, but the specific regulatory mechanism needs further investigation.

There are some limitations for this research. First of all, there are few data sets available for us to choose. The number of samples of DN patients is limited and clinical data of samples cannot be obtained. Secondly, when GSVA analysis is conducted, due to differences in sequencing genes obtained from different samples, all data sets cannot be used for analysis. Thirdly, the biomarkers identified in this study have not been experimentally verified, and the relevant mechanisms will be further investigated in future studies.

## 5 Conclusions

We determined that MMP2 is a gene closely related to DN fibrosis, which can effectively distinguish patients with DN from the control group. It is predicted that miR-106b-5p and miR-93-5p are upstream signals that regulate the expression of MMP2 and thereby produce a marked effect in DN.

## Data availability statement

Publicly available datasets were analyzed in this study. This data can be found here: https://www.ncbi.nlm.nih.gov/geo/, (Gene Expression Omnibus), (GSE142025,GSE96804).

## Author contributions

Research design, data processing and interpretation, statistical analysis and manuscript drafting were completed by DY. Research concept, data results check and manuscript knowledge revision were completed by ZG. Figures and diagrams drawing were completed by XZ. All authors contributed to the article and approved the submitted version.
